# Supplementary figures and images for: Different sound exposures causes alterations in stress-related serum indicators, behaviors, and cecal microbiota of green-shell egg-laying chickens under different stocking densities
Source: PeerJ. 2024 Nov 22;12:e18544. doi: 10.7717/peerj.18544 (PMC11587876; doi:10.7717/peerj.18544)

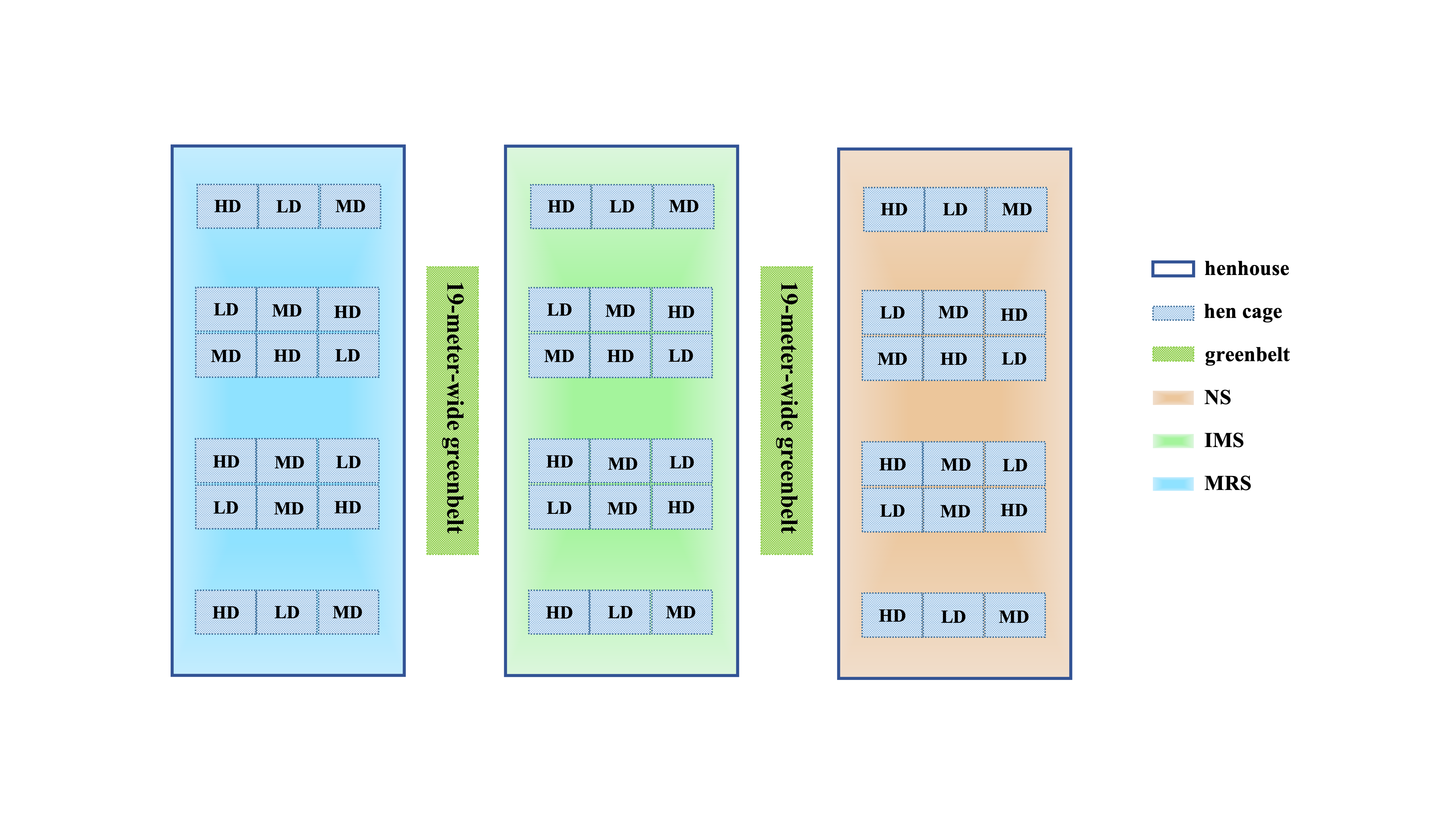

Supplement: Supplemental Information 1 — LD, low density (4 birds/cage); MD, medium density (7 birds/cage); high density (10 birds/cage); NS, natural sound; IMS, instrumental music sound; MRS, mixed road sound. Image credit: Shiwen Cao. [file peerj-12-18544-s001.png]

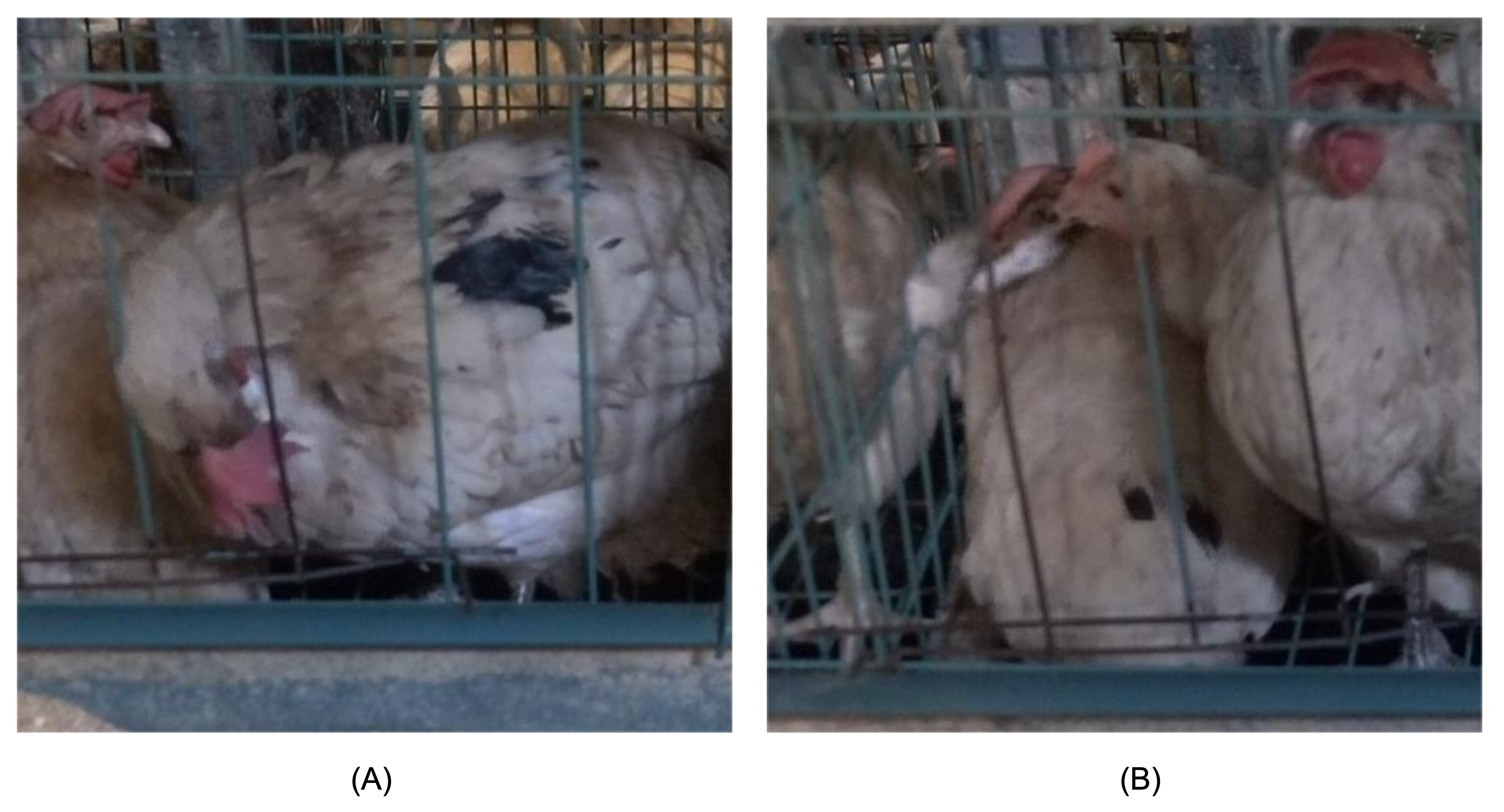

Supplement: Supplemental Information 2 — (A) Preening behavior; (B) Feather pecking behavior. Photo credit: Shiwen Cao. [file peerj-12-18544-s002.png]

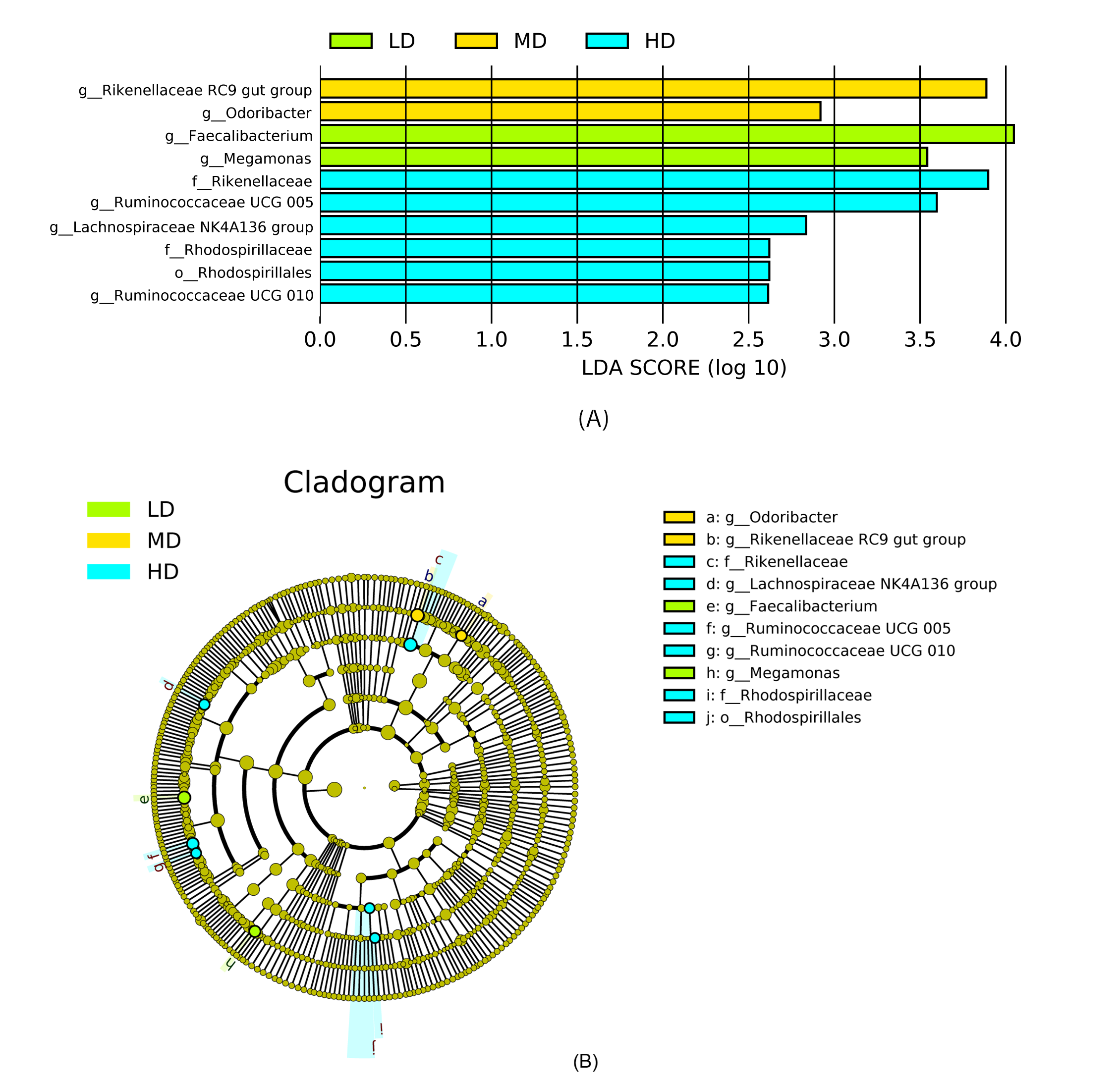

Supplement: Supplemental Information 3 — (A) Indicator bacteria identified by linear discriminant analysis (LDA) with a LDA score larger than 2.5. (B) Cladogram showing the phylogenetic distribution of the bacterial lineages. Circles indicate phylogenetic levels from phylum to genus and the diameter of each circle is proportional to the abundance of the group. Abbreviations: LD, low density; MD, medium density; HD, high density. Image credit: Shiwen Cao. [file peerj-12-18544-s003.png]

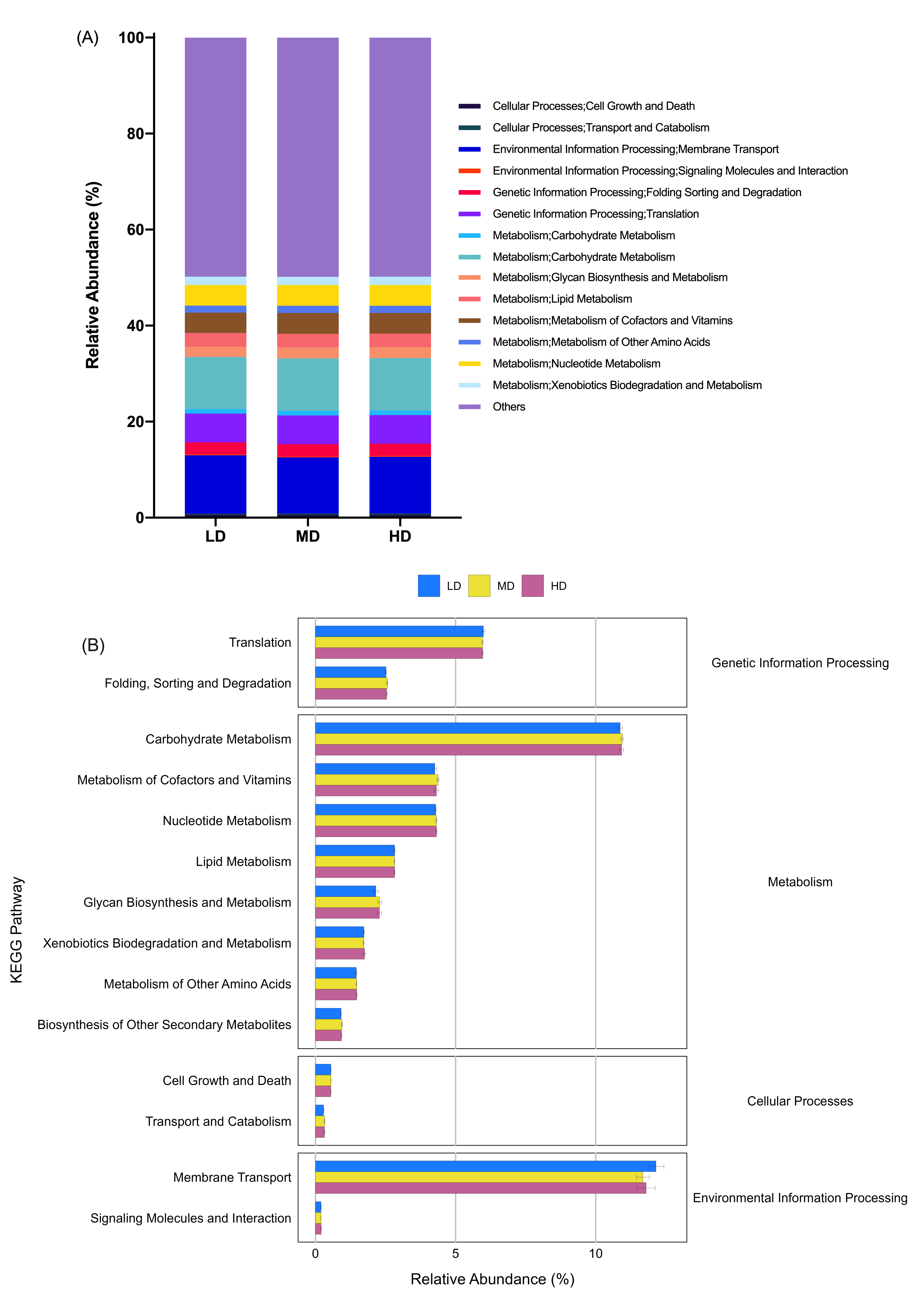

Supplement: Supplemental Information 4 — (A) KEGG annotation at level 2 under different stocking densities; (B) Differences in the enrichment of various KEGG pathways at level 2. LD, low density; MD, medium density; HD, high density. Data are presented as mean ± standard error of the mean (SEM). Image credit: Shiwen Cao. [file peerj-12-18544-s004.png]

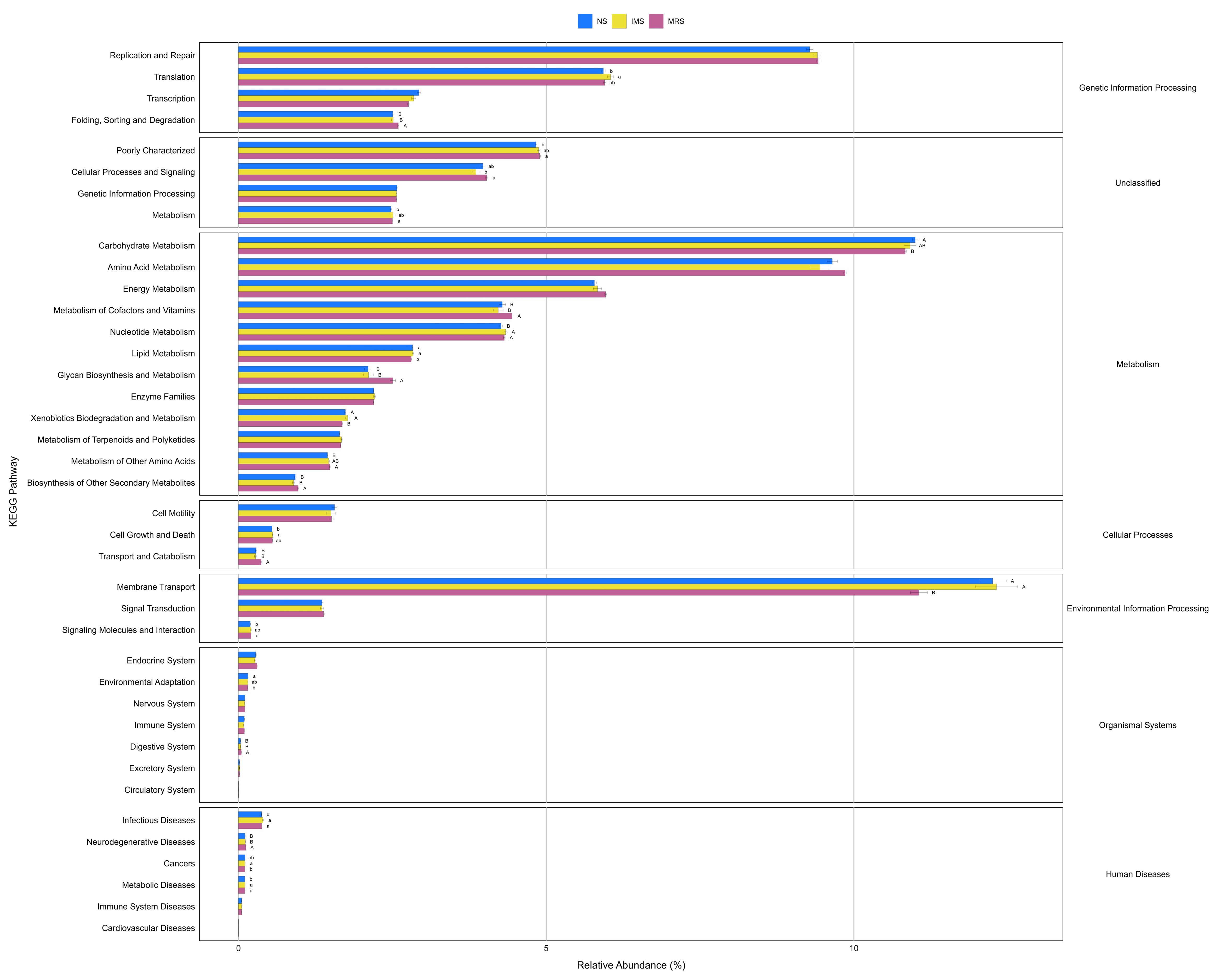

Supplement: Supplemental Information 5 — NS, natural sound; IMS, instrumental music; MRS, mixed road sound. Data are presented as mean ± standard error of the mean (SEM). Error bars indicate standard errors. Different low case letters and capital letters connect bar indicate significant differences (P ≤ 0.05, P ≤ 0.01, respectively). Image credit: Shiwen Cao. [file peerj-12-18544-s005.png]

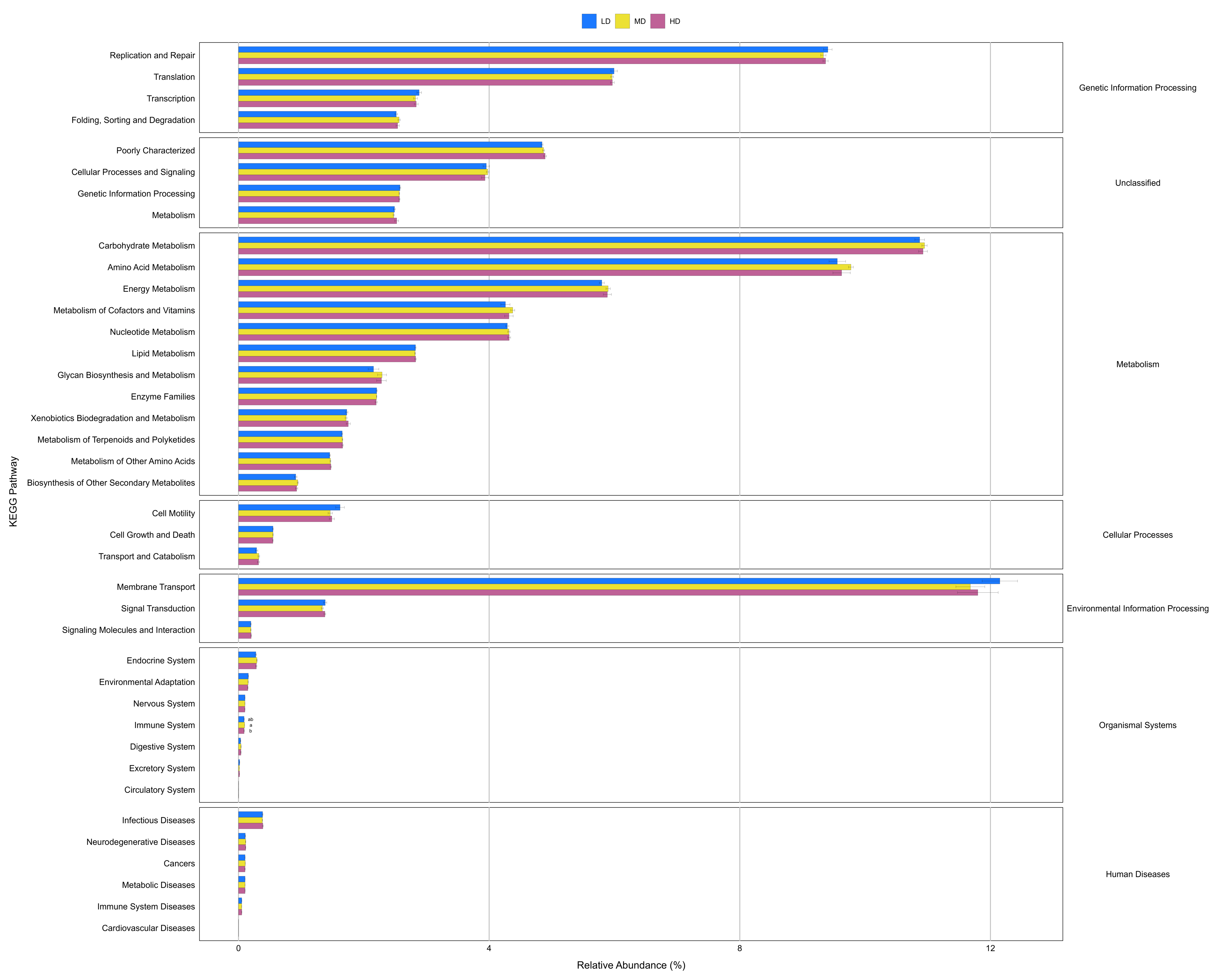

Supplement: Supplemental Information 6 — LD, low density; MD, medium density; HD, high density. Data are presented as mean ± standard error of the mean (SEM). Different low case letters connect bar indicate significant differences (P ≤ 0.05). Image credit: Shiwen Cao. [file peerj-12-18544-s006.png]
