# Supplementary material for: Different sound exposures causes alterations in stress-related serum indicators, behaviors, and cecal microbiota of green-shell egg-laying chickens under different stocking densities
Source: PeerJ. 2024 Nov 22;12:e18544. doi: 10.7717/peerj.18544 (PMC11587876; doi:10.7717/peerj.18544)
Supplement: Supplemental Information 7 — 1Trace mineral premix provided per kilogram of diet: Fe, 80 mg from iron sulfate; Mn, 100 mg from manganese oxide; Se, 0.15 mg from sodium selenite; I, 0.35 mg from ethylene diamine dihydroiodine; Cu, 8 mg from copper sulfate; Zn, 75 mg from zinc sulfate. Vitamin premix provide per kilogram of diet: VA 5000 IU, VD3 2500 IU, VE 18.75 mg, VK3 2.65 mg, VB1 2 mg, VB2 6 mg, VB12 0.025 mg, biotin 0.0325 mg, folic acid 1.25 mg, pantothenic acid 12 mg, nicotinic acid 50 mg. 2Crude protein is a measured value and the others are calculated values. [file peerj-12-18544-s007.doc]

**Table S1:**

**Composition of the standard layer diet used in this experiment.**

| Ingredients (%） | Content |
| --- | --- |
| Corn | 61.00 |
| Soybean meal | 20.00 |
| Shell powder | 8.50 |
| Trace mineral and vitamin premix^1^ | 5.00 |
| Lard | 2.50 |
| Corn germ meal | 3.00 |
| Total | 100 |
| Nutrient levels |  |
| Metabolizible energy (MJ/kg) | 11.30 |
| Crude protein (%)^2^ | 16.50 |
| Methionine (%) | 0.28 |
| Other amino acids | 0.55 |
| Ca (%) | 3.50 |
| P (%) | 0.35 |

**Notes:**

^1^Trace mineral premix provided per kilogram of diet: Fe, 80 mg from iron sulfate; Mn, 100 mg from manganese oxide; Se, 0.15 mg from sodium selenite; I, 0.35 mg from ethylene diamine dihydroiodine; Cu, 8 mg from copper sulfate; Zn, 75 mg from zinc sulfate. Vitamin premix provide per kilogram of diet: V_A_ 5000 IU, V_D3_ 2500 IU, V_E_ 18.75 mg, V_K3_ 2.65 mg, V_B1_ 2 mg, V_B2_ 6 mg, V_B12_ 0.025 mg, biotin 0.0325 mg, folic acid 1.25 mg, pantothenic acid 12 mg, nicotinic acid 50 mg.

^2^Crude protein is a measured value and the others are calculated values.
