# Supplementary material for: Different sound exposures causes alterations in stress-related serum indicators, behaviors, and cecal microbiota of green-shell egg-laying chickens under different stocking densities
Source: PeerJ. 2024 Nov 22;12:e18544. doi: 10.7717/peerj.18544 (PMC11587876; doi:10.7717/peerj.18544)
Supplement: Supplemental Information 8 — NS, natural sound; IMS, instrumental music; MRS, mixed road sound; LD, low density; MD, medium density; HD, high density; NL, NS + LD; NM, NS + MD; NH, NS + HD; IML, IMS + LD; IMM, IMS + MD; IMH, IMS + HD; MRL, MRS + LD; MRM, MRS + MD; MRH, MRS + HD. Data are presented as mean ± standard error of the mean (SEM). a,bMeans with different low case letters within a column indicate significant differences (P ≤ 0.05). A,BMeans with different capital letters within a column indicate very significant differences (P ≤ 0.01). [file peerj-12-18544-s008.doc]

**Table S2:**

**Effects of different sound sources and stocking densities on the alpha diversity of bacterial communities in cecal digesta (n = 6).**

|  |  | Goods coverage | Chao1 | Observed species | PD whole tree | Shannon | Simpson |
| --- | --- | --- | --- | --- | --- | --- | --- |
| Group | NL | 0.9967±0.0002 | 801.61±36.29 | 682.50±38.14 | 44.31±1.05 | 7.03±0.17 | 0.981±0.003 |
|  | NM | 0.9969±0.0001 | 807.67±25.69 | 698.83±29.05 | 44.70±1.86 | 7.26±0.10 | 0.984±0.001 |
|  | NH | 0.9971±0.0001 | 799.96±20.78 | 713.17±17.37 | 44.41±0.76 | 7.28±0.06 | 0.984±0.001 |
|  | IML | 0.9966±0.0001 | 791.34±20.21 | 675.80±21.48 | 43.30±1.33 | 6.84±0.16 | 0.970±0.008 |
|  | IMM | 0.9969±0.0001 | 757.54±4.64 | 651.83±9.02 | 41.81±0.48 | 6.99±0.02 | 0.981±0.001 |
|  | IMH | 0.9965±0.0001 | 746.50±44.85 | 659.08±29.59 | 42.66±2.90 | 6.32±0.49 | 0.913±0.040 |
|  | MRL | 0.9967±0.0001 | 799.98±13.01 | 697.33±15.10 | 43.48±0.82 | 7.06±0.05 | 0.979±0.001 |
|  | MRM | 0.9968±0.0002 | 794.34±32.96 | 683.17±25.46 | 43.99±1.56 | 7.12±0.10 | 0.980±0.003 |
|  | MRH | 0.9967±0.0002 | 837.00±26.16 | 713.50±10.70 | 44.83±0.70 | 7.07±0.05 | 0.976±0.002 |
| Main effect | |  | | | | | |
| Sound  (S) | NS | 0.9969±0.0001 | 803.08±15.39 | 698.17±16.25 | 44.47±0.71 | 7.19±0.07^A^ | 0.983±0.001^a^ |
|  | IMS | 0.9967±0.0001 | 765.13±16.15 | 662.24±12.04 | 42.59±1.02 | 6.72±0.17^B^ | 0.955±0.015^b^ |
|  | MRS | 0.9967±0.0001 | 810.44±14.54 | 698.00±10.30 | 44.10±0.61 | 7.08±0.04^A^ | 0.978±0.001^a^ |
| Density  (D) | LD | 0.9967±0.0001 | 797.65±13.67 | 685.21±14.66 | 43.70±0.60 | 6.98±0.08 | 0.977±0.003 |
|  | MD | 0.9969±0.0001 | 786.52±14.13 | 677.94±13.29 | 43.50±0.83 | 7.12±0.05 | 0.982±0.001 |
|  | HD | 0.9968±0.0001 | 794.49±19.69 | 695.25±12.85 | 43.97±0.99 | 6.89±0.18 | 0.958±0.015 |
| *P* values | |  |  |  |  |  |  |
| Sound | | 0.114 | 0.107 | 0.111 | 0.257 | 0.009 | 0.034 |
| Density | | 0.188 | 0.878 | 0.668 | 0.926 | 0.323 | 0.095 |
| S×D | | 0.139 | 0.621 | 0.842 | 0.931 | 0.239 | 0.077 |

**Notes:**

NS, natural sound; IMS, instrumental music; MRS, mixed road sound; LD, low density; MD, medium density; HD, high density; NL, NS + LD; NM, NS + MD; NH, NS + HD; IML, IMS + LD; IMM, IMS + MD; IMH, IMS + HD; MRL, MRS + LD; MRM, MRS + MD; MRH, MRS + HD. Data are presented as mean ± standard error of the mean (SEM).

^a,b^Means with different low case letters within a column indicate significant differences (*P* ≤ 0.05).

^A,B^Means with different capital letters within a column indicate very significant differences (*P* ≤ 0.01).
