# Supplementary material for: Different sound exposures causes alterations in stress-related serum indicators, behaviors, and cecal microbiota of green-shell egg-laying chickens under different stocking densities
Source: PeerJ. 2024 Nov 22;12:e18544. doi: 10.7717/peerj.18544 (PMC11587876; doi:10.7717/peerj.18544)
Supplement: Supplemental Information 9 — NS, natural sound; IMS, instrumental music; MRS, mixed road sound; LD, low density; MD, medium density; HD, high density; NL, NS + LD; NM, NS + MD; NH, NS + HD; IML, IMS + LD; IMM, IMS + MD; IMH, IMS + HD; MRL, MRS + LD; MRM, MRS + MD; MRH, MRS + HD. Data are presented as mean ± standard error of the mean (SEM). A,BMeans with different capital letters within a column indicate very significant differences (P ≤ 0.01). [file peerj-12-18544-s009.docx]

**Table S3:**

**The frequency (n) and duration (s) of drinking behavior (n = 15).**

|  | | Frequency | | | | Duration | | | |
| --- | --- | --- | --- | --- | --- | --- | --- | --- | --- |
|  |  | Day 3 | Day 12 | Day 24 | Day 3 | | Day 12 | Day 24 |  |
| Group | NL | 1.93±0.54 | 4.67±1.12 | 4.27±1.22 | 18.33±6.55 | | 63.67±19.02 | 55.67±31.65 |  |
|  | NM | 2.93±1.02 | 1.07±0.33 | 2.67±0.94 | 52.00±24.68 | | 9.73±2.97 | 46.47±18.43 |  |
|  | NH | 1.20±0.44 | 1.27±0.42 | 1.47±0.58 | 12.33±6.14 | | 14.73±6.17 | 11.53±4.58 |  |
|  | IML | 2.80±1.13 | 5.87±1.09 | 3.40±0.79 | 30.80±13.24 | | 34.13±5.65 | 22.13±5.92 |  |
|  | IMM | 3.40±1.00 | 2.93±0.81 | 2.67±0.57 | 42.73±23.35 | | 37.00±13.45 | 22.20±6.43 |  |
|  | IMH | 2.27±1.01 | 1.33±0.55 | 2.33±1.10 | 27.00±12.83 | | 10.27±3.66 | 35.00±21.25 |  |
|  | MRL | 4.67±1.31 | 2.67±0.93 | 3.07±0.71 | 39.53±11.28 | | 31.20±10.82 | 26.27±7.15 |  |
|  | MRM | 1.67±0.65 | 1.80±0.82 | 0.47±0.19 | 17.27±7.94 | | 17.67±10.48 | 2.07±0.88 |  |
|  | MRH | 0.80±0.37 | 1.33±1.13 | 1.40±0.40 | 6.73±3.76 | | 20.00±18.60 | 9.93±4.02 |  |
| Main effect | |  |  |  |  | |  |  |  |
| Sound (S) | NS | 2.02±0.42 | 2.33±0.48 | 2.80±0.56 | 27.56±8.95 | | 29.38±7.54 | 37.89±12.36 |  |
|  | IMS | 2.82±0.60 | 3.38±0.56 | 2.80±0.48 | 33.51±9.74 | | 27.13±5.22 | 26.44±7.54 |  |
|  | MRS | 2.38±0.55 | 1.93±0.55 | 1.64±0.32 | 21.18±5.09 | | 22.96±7.85 | 12.76±3.09 |  |
| Density (D) | LD | 3.13±0.62 | 4.40±0.63^A^ | 3.58±0.53^A^ | 29.56±6.19 | | 43.00±7.68^A^ | 34.69±10.98 |  |
|  | MD | 2.67±0.52 | 1.93±0.41^B^ | 1.93±0.39^B^ | 37.33±11.58 | | 21.47±5.90^B^ | 23.58±6.93 |  |
|  | HD | 1.42±0.39 | 1.31±0.43^B^ | 1.73±0.43^B^ | 15.36±4.96 | | 15.00±6.52^B^ | 18.82±7.40 |  |
| *P* value | |  |  |  |  | |  |  |  |
| Sound | | 0.545 | 0.104 | 0.119 | 0.563 | | 0.789 | 0.117 |  |
| Density | | 0.055 | < 0.001 | 0.008 | 0.156 | | 0.010 | 0.404 |  |
| S×D | | 0.133 | 0.288 | 0.556 | 0.363 | | 0.114 | 0.287 |  |

**Notes:**

NS, natural sound; IMS, instrumental music; MRS, mixed road sound; LD, low density; MD, medium density; HD, high density; NL, NS + LD; NM, NS + MD; NH, NS + HD; IML, IMS + LD; IMM, IMS + MD; IMH, IMS + HD; MRL, MRS + LD; MRM, MRS + MD; MRH, MRS + HD. Data are presented as mean ± standard error of the mean (SEM).

^A,B^Means with different capital letters within a column indicate very significant differences (*P* ≤ 0.01).
