# Supplementary material for: Different sound exposures causes alterations in stress-related serum indicators, behaviors, and cecal microbiota of green-shell egg-laying chickens under different stocking densities
Source: PeerJ. 2024 Nov 22;12:e18544. doi: 10.7717/peerj.18544 (PMC11587876; doi:10.7717/peerj.18544)
Supplement: Supplemental Information 10 — NS, natural sound; IMS, instrumental music; MRS, mixed road sound; LD, low density; MD, medium density; HD, high density; NL, NS + LD; NM, NS + MD; NH, NS + HD; IML, IMS + LD; IMM, IMS + MD; IMH, IMS + HD; MRL, MRS + LD; MRM, MRS + MD; MRH, MRS + HD. Data are presented as mean ± standard error of the mean (SEM). a,bMeans with different low case letters within a column indicate significant differences (P ≤ 0.05). [file peerj-12-18544-s010.docx]

**Table S4:**

**The frequency (n) and duration (s) of feeding behavior (n = 15).**

|  | | Frequency | | | Duration | | |
| --- | --- | --- | --- | --- | --- | --- | --- |
|  |  | Day 3 | Day 12 | Day 24 | Day 3 | Day 12 | Day 24 |
| Group | NL | 9.60±1.77 | 10.40±1.42 | 9.00±1.20 | 229.33±50.97 | 257.93±32.04 | 181.87±26.46 |
|  | NM | 8.40±1.34 | 7.00±1.14 | 5.87±1.31 | 180.27±33.80 | 189.60±39.23 | 155.20±41.07 |
|  | NH | 6.73±1.41 | 6.40±1.13 | 10.07±2.30 | 129.60±32.45 | 167.47±41.67 | 175.87±45.35 |
|  | IML | 5.27±1.27 | 10.20±1.53 | 7.00±0.81 | 112.53±30.34 | 235.53±37.26 | 173.33±32.81 |
|  | IMM | 9.40±1.21 | 9.00±1.47 | 8.20±1.78 | 202.53±42.50 | 256.33±54.25 | 215.60±39.89 |
|  | IMH | 8.60±1.05 | 8.27±1.37 | 9.67±1.83 | 163.60±34.35 | 150.40±32.67 | 246.93±60.82 |
|  | MRL | 8.60±1.98 | 6.40±1.70 | 8.40±1.57 | 187.40±41.43 | 204.20±47.07 | 176.00±46.56 |
|  | MRM | 7.80±1.28 | 9.93±1.22 | 4.67±1.33 | 144.27±31.01 | 316.87±47.81 | 98.53±30.52 |
|  | MRH | 8.93±1.78 | 5.27±1.28 | 7.20±1.57 | 196.27±53.73 | 155.47±53.52 | 92.73±24.54 |
| Main effect | |  |  |  |  |  |  |
| Sound (S) | NS | 8.24±0.88 | 7.93±0.75 | 8.31±0.98 | 179.73±23.37 | 205.00±22.14 | 170.98±21.78^ab^ |
|  | IMS | 7.76±0.72 | 9.16±0.83 | 8.29±0.89 | 159.56±21.10 | 214.09±24.90 | 211.96±26.38^a^ |
|  | MRS | 8.44±0.96 | 7.20±0.85 | 6.76±0.88 | 175.98±24.53 | 225.51±29.75 | 122.42±20.62^b^ |
| Density (D) | LD | 7.82±1.00 | 9.00±0.92 | 8.13±0.71 | 176.42±24.66 | 232.56±22.41^a^ | 177.07±20.46 |
|  | MD | 8.53±0.73 | 8.64±0.75 | 6.24±0.87 | 175.69±20.68 | 254.27±27.91^a^ | 156.44±22.32 |
|  | HD | 8.09±0.83 | 6.64±0.74 | 8.98±1.10 | 163.16±23.66 | 157.78±24.54^b^ | 171.84±27.65 |
| *P* value | |  |  |  |  |  |  |
| Sound | | 0.843 | 0.217 | 0.385 | 0.804 | 0.846 | 0.026 |
| Density | | 0.839 | 0.082 | 0.098 | 0.900 | 0.020 | 0.808 |
| S×D | | 0.192 | 0.139 | 0.485 | 0.174 | 0.315 | 0.386 |

**Notes:**

NS, natural sound; IMS, instrumental music; MRS, mixed road sound; LD, low density; MD, medium density; HD, high density; NL, NS + LD; NM, NS + MD; NH, NS + HD; IML, IMS + LD; IMM, IMS + MD; IMH, IMS + HD; MRL, MRS + LD; MRM, MRS + MD; MRH, MRS + HD. Data are presented as mean ± standard error of the mean (SEM).

^a,b^Means with different low case letters within a column indicate significant differences (*P* ≤ 0.05).
