# Supplementary material for: Different sound exposures causes alterations in stress-related serum indicators, behaviors, and cecal microbiota of green-shell egg-laying chickens under different stocking densities
Source: PeerJ. 2024 Nov 22;12:e18544. doi: 10.7717/peerj.18544 (PMC11587876; doi:10.7717/peerj.18544)
Supplement: Supplemental Information 11 — NS, natural sound; IMS, instrumental music; MRS, mixed road sound; LD, low density; MD, medium density; HD, high density; NL, NS + LD; NM, NS + MD; NH, NS + HD; IML, IMS + LD; IMM, IMS + MD; IMH, IMS + HD; MRL, MRS + LD; MRM, MRS + MD; MRH, MRS + HD. Data are presented as mean ± standard error of the mean (SEM). a,bMeans with different low case letters within a column indicate significant differences (P ≤ 0.05). A,BMeans with different capital letters within a column indicate very significant differences (P ≤ 0.01). [file peerj-12-18544-s011.docx]

**Table S5:**

**The frequency (n) and duration (s) of lying behavior (n = 15).**

|  | | Frequency | | | Duration | | |
| --- | --- | --- | --- | --- | --- | --- | --- |
|  |  | Day 3 | Day 12 | Day 24 | Day 3 | Day 12 | Day 24 |
| Group | NL | 0.53±0.34^b^ | 1.07±0.46 | 3.20±0.64 | 10.67±8.61^b^ | 61.93±34.41 | 88.13±22.90 |
|  | NM | 0.47±0.17^ab^ | 1.27±0.41 | 2.87±0.63 | 4.00±1.73^ab^ | 41.33±17.53 | 89.73±27.75 |
|  | NH | 1.53±0.41^ab^ | 1.73±0.53 | 3.07±0.74 | 23.67±7.17^ab^ | 40.73±13.56 | 87.40±26.99 |
|  | IML | 2.00±0.58^a^ | 0.87±0.44 | 3.47±1.10 | 64.67±21.84^a^ | 20.87±10.95 | 79.27±33.16 |
|  | IMM | 1.73±0.61^ab^ | 2.40±0.73 | 3.93±0.97 | 46.00±24.48^a^ | 92.67±39.00 | 117.13±34.21 |
|  | IMH | 0.60±0.16^b^ | 3.53±0.74 | 2.80±0.60 | 6.80±2.22^b^ | 78.33±20.44 | 61.67±15.50 |
|  | MRL | 0.80±0.37^b^ | 0.87±0.26 | 1.73±0.72 | 15.27±7.48^b^ | 21.87±7.07 | 40.93±22.19 |
|  | MRM | 1.67±0.44^ab^ | 0.47±0.19 | 1.53±0.39 | 29.00±8.63^ab^ | 10.87±7.41 | 29.47±9.13 |
|  | MRH | 1.13±0.49^ab^ | 1.27±0.45 | 1.73±0.33 | 26.07±17.05^ab^ | 31.40±15.12 | 32.40±9.87 |
| Main effect | |  |  |  |  |  |  |
| Sound (S) | NS | 0.84±0.20 | 1.36±0.27^B^ | 3.04±0.38^A^ | 12.78±3.89 | 48.00±13.41^ab^ | 88.42±14.65^A^ |
|  | IMS | 1.44±0.29 | 2.27±0.40^A^ | 3.40±0.52^A^ | 39.16±11.31 | 63.96±15.50^a^ | 86.02±16.69^A^ |
|  | MRS | 1.20±0.25 | 0.87±0.19^B^ | 1.67±0.29^B^ | 23.44±6.74 | 21.38±6.08^b^ | 34.27±8.48^B^ |
| Density (D) | LD | 1.11±0.27 | 0.93±0.22^B^ | 2.80±0.49 | 30.20±8.83 | 34.89±12.33 | 69.44±15.30 |
|  | MD | 1.29±0.27 | 1.38±0.30^AB^ | 2.78±0.43 | 26.33±8.86 | 48.29±15.02 | 78.78±15.66 |
|  | HD | 1.09±0.22 | 2.18±0.36^A^ | 2.53±0.34 | 18.84±6.20 | 50.16±9.87 | 60.49±11.16 |
| *P* value | |  |  |  |  |  |  |
| Sound | | 0.222 | 0.003 | 0.009 | 0.058 | 0.050 | 0.009 |
| Density | | 0.818 | 0.010 | 0.881 | 0.577 | 0.632 | 0.649 |
| S×D | | 0.022 | 0.140 | 0.884 | 0.042 | 0.167 | 0.729 |

**Notes:**

NS, natural sound; IMS, instrumental music; MRS, mixed road sound; LD, low density; MD, medium density; HD, high density; NL, NS + LD; NM, NS + MD; NH, NS + HD; IML, IMS + LD; IMM, IMS + MD; IMH, IMS + HD; MRL, MRS + LD; MRM, MRS + MD; MRH, MRS + HD. Data are presented as mean ± standard error of the mean (SEM).

^a,b^Means with different low case letters within a column indicate significant differences (*P* ≤ 0.05).

^A,B^Means with different capital letters within a column indicate very significant differences (*P* ≤ 0.01).
