# Supplementary material for: Different sound exposures causes alterations in stress-related serum indicators, behaviors, and cecal microbiota of green-shell egg-laying chickens under different stocking densities
Source: PeerJ. 2024 Nov 22;12:e18544. doi: 10.7717/peerj.18544 (PMC11587876; doi:10.7717/peerj.18544)
Supplement: Supplemental Information 12 — NS, natural sound; IMS, instrumental music; MRS, mixed road sound; LD, low density; MD, medium density; HD, high density; NL, NS + LD; NM, NS + MD; NH, NS + HD; IML, IMS + LD; IMM, IMS + MD; IMH, IMS + HD; MRL, MRS + LD; MRM, MRS + MD; MRH, MRS + HD. Data are presented as mean ± standard error of the mean (SEM). a,bMeans with different low case letters within a column indicate significant differences (P ≤ 0.05). A,B,CMeans with different capital letters within a column indicate very significant differences (P ≤ 0.01). [file peerj-12-18544-s012.docx]

**Table S6:**

**The frequency (n) and duration (s) of standing behavior (n = 15).**

|  | | Frequency | | | Duration | | |
| --- | --- | --- | --- | --- | --- | --- | --- |
|  |  | Day 3 | Day 12 | Day 24 | Day 3 | Day 12 | Day 24 |
| Group | NL | 25.27±1.35 | 21.27±1.52 | 26.93±1.49 | 371.73±30.58 | 312.53±31.19 | 351.13±27.16 |
|  | NM | 27.47±2.16 | 25.27±1.63 | 26.73±1.98 | 451.13±29.09 | 449.13±31.97 | 445.87±38.67 |
|  | NH | 27.00±1.26 | 27.53±1.41 | 28.87±1.52 | 529.33±26.39 | 483.67±36.57 | 424.93±26.09 |
|  | IML | 25.87±1.29 | 27.87±1.22 | 34.73±3.13 | 347.07±23.36 | 363.47±27.17 | 360.60±29.16 |
|  | IMM | 26.33±1.73 | 25.60±2.01 | 29.53±2.30 | 349.40±35.42 | 327.73±30.99 | 357.07±30.99 |
|  | IMH | 29.13±1.55 | 29.27±2.11 | 31.67±2.63 | 444.13±30.62 | 466.87±31.10 | 372.67±38.60 |
|  | MRL | 26.27±1.55 | 25.67±1.82 | 33.80±2.28 | 309.27±40.42 | 373.13±38.36 | 417.80±35.94 |
|  | MRM | 26.00±1.60 | 27.80±3.51 | 39.93±2.15 | 486.33±32.12 | 366.87±31.65 | 531.87±31.63 |
|  | MRH | 28.40±1.23 | 24.47±1.66 | 38.07±2.14 | 466.27±36.68 | 505.60±43.52 | 585.13±22.82 |
| Main effect | |  |  |  |  |  |  |
| Sound (S) | NS | 26.58±0.94 | 24.69±0.94 | 27.51±0.96^C^ | 450.73±18.89^a^ | 415.11±21.85 | 407.31±18.62^B^ |
|  | IMS | 27.11±0.89 | 27.58±1.05 | 31.98±1.56^B^ | 380.20±18.35^b^ | 386.02±19.02 | 363.44±18.74^B^ |
|  | MRS | 26.89±0.84 | 25.98±1.41 | 37.27±1.29^A^ | 420.62±23.83^ab^ | 415.20±23.58 | 511.60±20.22^A^ |
| Density (D) | LD | 25.80±0.79 | 24.93±0.96 | 31.82±1.45 | 342.69±18.58^B^ | 349.71±18.81^B^ | 376.51±18.03^B^ |
|  | MD | 26.60±1.05 | 26.22±1.43 | 32.07±1.48 | 428.96±20.22^A^ | 381.24±19.36^B^ | 444.93±21.96^A^ |
|  | HD | 28.18±0.77 | 27.09±1.03 | 32.87±1.34 | 479.91±18.59^A^ | 485.38±21.24^A^ | 460.91±21.71^A^ |
| *P* value | |  |  |  |  |  |  |
| Sound | | 0.914 | 0.205 | < 0.001 | 0.028 | 0.481 | < 0.001 |
| Density | | 0.165 | 0.409 | 0.836 | < 0.001 | < 0.001 | 0.003 |
| S×D | | 0.826 | 0.170 | 0.148 | 0.089 | 0.111 | 0.114 |

**Notes:**

NS, natural sound; IMS, instrumental music; MRS, mixed road sound; LD, low density; MD, medium density; HD, high density; NL, NS + LD; NM, NS + MD; NH, NS + HD; IML, IMS + LD; IMM, IMS + MD; IMH, IMS + HD; MRL, MRS + LD; MRM, MRS + MD; MRH, MRS + HD. Data are presented as mean ± standard error of the mean (SEM).

^a,b^Means with different low case letters within a column indicate significant differences (*P* ≤ 0.05).

^A,B,C^Means with different capital letters within a column indicate very significant differences (*P* ≤ 0.01).
